# Mechanisms of resistance to trastuzumab emtansine (T-DM1) in HER2-positive breast cancer

**DOI:** 10.1038/s41416-019-0635-y

**Published:** 2019-12-16

**Authors:** Francis W. Hunter, Hilary R. Barker, Barbara Lipert, Françoise Rothé, Géraldine Gebhart, Martine J. Piccart-Gebhart, Christos Sotiriou, Stephen M. F. Jamieson

**Affiliations:** 1 Auckland Cancer Society Research Centre, University of Auckland, Auckland, New Zealand; 2 Maurice Wilkins Centre for Molecular Biodiscovery, University of Auckland, Auckland, New Zealand; 3 Institut Jules Bordet, Universite Libre de Bruxelles, Brussels, Belgium; 4 Department of Pharmacology and Clinical Pharmacology, Faculty of Medical and Health Sciences, University of Auckland, Auckland, New Zealand

**Keywords:** Breast cancer, Cancer therapeutic resistance

## Abstract

The HER2-targeted antibody–drug conjugate trastuzumab emtansine (T-DM1) is approved for the treatment of metastatic, HER2-positive breast cancer after prior trastuzumab and taxane therapy, and has also demonstrated efficacy in the adjuvant setting in incomplete responders to neoadjuvant therapy. Despite its objective activity, intrinsic and acquired resistance to T-DM1 remains a major clinical challenge. T-DM1 mediates its activity in a number of ways, encompassing HER2 signalling blockade, Fc-mediated immune response and payload-mediated microtubule poisoning. Resistance mechanisms relating to each of these features have been demonstrated, and we outline the findings of these studies in this review. In our overview of the substantial literature on T-DM1 activity and resistance, we conclude that the T-DM1 resistance mechanisms most strongly supported by the experimental data relate to dysfunctional intracellular metabolism of the construct and subversion of DM1-mediated cell killing. Loss of dependence on signalling initiated by HER2–HER2 homodimers is not substantiated as a resistance mechanism by clinical or experimental studies, and the impact of EGFR expression and tumour immunological status requires further investigation. These findings are instructive with respect to strategies that might overcome T-DM1 resistance, including the use of second-generation anti-HER2 antibody–drug conjugates that deploy alternative linker-payload chemistries.

## Background

**Fig. 1:**
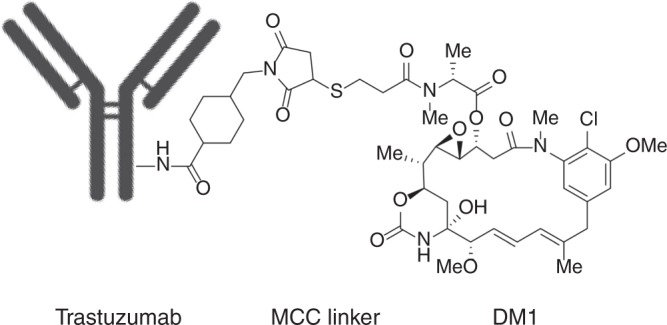
Structure of T-DM1. T-DM1 is comprised of the monoclonal antibody trastuzumab conjugated via a non-cleavable MCC thioether linker to 3–3.6 moieties of the potent tubulin polymerisation inhibitor mertansine (DM1).

Trastuzumab emtansine (T-DM1, Kadcyla®) is an antibody–drug conjugate (ADC) comprised of the humanised, monoclonal, anti-HER2 antibody trastuzumab conjugated via a non-cleavable maleimidomethyl cyclohexane-1-carboxylate (MCC) thioether linker to the highly potent cytotoxin DM1 (Fig. 1). DM1 is a derivative of the naturally occurring maytansinoid toxin, which inhibits tubulin polymerisation and induces death in proliferative cells. DM1 has a narrow therapeutic window for oncology, but its linkage to trastuzumab, with an average of 3.5 linker-payload molecules per antibody (drug–antibody ratio [DAR] of 3.5), selectively targets the cytotoxin to malignant cells that overexpress the HER2 receptor tyrosine kinase (RTK), thereby widening its therapeutic window.^1^ T-DM1 gained European Medicines Agency (EMA) and Food and Drug Administration (FDA) approval in 2013 for the treatment, as a single agent, of HER2-positive metastatic breast cancer in patients who have progressed despite prior therapy with trastuzumab and a taxane (paclitaxel or docetaxel).

Although treatment with T-DM1 significantly improves outcomes in HER2-positive breast cancer, a substantial fraction of patients are refractory to T-DM1 and, as with all drugs currently indicated for metastatic breast cancer, acquired resistance to T-DM1 almost always presents in initial responders.^2,3^ To address this problem of resistance, considerable effort has been undertaken to investigate the complex mechanisms of action of T-DM1 and to understand how evasion of these mechanisms can lead to treatment failure. This review summarises the clinical activity of T-DM1 in the treatment of HER2-positive breast cancer, outlines the multiple mechanisms of action of T-DM1 and describes various resistance mechanisms that relate to the actions of T-DM1. By presenting the preclinical and clinical evidence that underpins each proposed resistance mechanism, we identify the mechanisms that are most substantiated to contribute to T-DM1 resistance and discuss how understanding these resistance mechanisms can assist novel strategies, including second-generation HER2-targeting ADCs, to overcome T-DM1 resistance.

## Clinical activity of T-DM1

The efficacy of T-DM1 as an adjuvant, neoadjuvant, first-line and second-line metastatic therapy for HER2-positive breast cancer has been evaluated in five randomised Phase 3 trials (Table 1).

**Table 1:** Summary of Phase 3 clinical trial data for T-DM1.

Trial	Endpoint	Experimental therapy	Control arm	Hazard ratio (95% CI)	Reference
*EMILIA (HER2-positive advanced breast cancer previously treated with trastuzumab and a taxane)*					^2^
		T-DM1 (*n* = 495)	Lapatinib + capecitabine (*n* = 496)		
	Overall survival	30.9 months	25.1 months	0.65 (0.55–0.77)	
	Progression-free survival	9.6 months	6.4 months	0.68 (0.55–0.86)	
	Grade ≥ 3 adverse events	48%	60%		
*TH3RESA (HER2-positive advanced breast cancer previously treated with both trastuzumab and lapatinib in the advanced setting and a taxane in any setting)*					^3^
		T-DM1 (*n* = 404)	Physician’s choice^a^ (*n* = 198)		
	Overall survival	22.7 months	15.8 months	0.68 (0.54–0.85)	
	Progression-free survival	6.2 months	3.3 months	0.53 (0.42–0.66)	
	Grade ≥ 3 adverse events	40%	47%		
	Treatment exposure-adjusted rate of grade ≥ 3 adverse events	123.6/100 patient years	278.4/100 patient years		
*MARIANNE (HER2-positive advanced breast cancer or previously untreated metastatic breast cancer)*					^5^
		T-DM1 (*n* = 367)	Trastuzumab + taxane (*n* = 365)		
	Progression-free survival	14.1 months	13.7 months	0.91 (0.73–1.13)^b^	
	Grade ≥ 3 adverse events	45.4%	54.1%		
	Time to decrease in HRQOL	7.7 months	3.6 months		
		T-DM1 + pertuzumab (*n* = 363)	Trastuzumab + taxane (*n* = 365)		
	Progression-free survival	15.2 months	13.7 months	0.87 (0.69–1.08)^b^	
	Grade ≥ 3 adverse events	46.2%	54.1%		
	Time to decrease in HRQOL	9.0 months	3.6 months		
*KRISTINE (HER2-positive breast cancer in the neoadjuvant setting)*					^6,7^
		T-DM1 + pertuzumab (*n* = 223)	Trastuzumab, pertuzumab, docetaxel + carboplatin (*n* = 221)		
	Pathological complete response	44.3%	55.7%		
	Grade ≥ 3 adverse events	13%	64%		
*KATHERINE (HER2-positive early breast cancer with residual invasive disease at surgery after neoadjuvant therapy with trastuzumab and a taxane)*					^11^
		T-DM1 (*n* = 743)	Trastuzumab (*n* = 743)		
	Invasive disease-free survival	87.8%	77.8%	0.50 (0.39–0.64)	
	Freedom from distant recurrence	89.5%	83.7%	0.60 (0.45–0.79)	
	Overall survival	94.3%	92.5%	0.70 (0.47–1.05)	
	Grade ≥ 3 adverse events	15.4%	25.7%		

HRQOL health-related quality of life

^a^Physician’s choice included chemotherapy, hormonal therapy and HER2-directed therapy

^b^97.5% confidence interval (CI)

### The EMILIA study

The initial regulatory approval of T-DM1 was informed by the EMILIA study, in which HER2-positive metastatic breast cancer previously treated with trastuzumab and a taxane demonstrated improvements in median overall survival (OS) and progression-free survival (PFS) in the T-DM1-treated group compared with the reference arm, which was treated with lapatinib and capecitabine, the standard second-line therapy for metastatic breast cancer at that time.^2^ Notably, in this study, the most commonly occurring adverse events in the T-DM1 cohort were changes in clinical laboratory test results (e.g. thrombocytopenia and elevated levels of liver enzymes), in contrast with symptomatic adverse events (e.g. diarrhoea) observed in patients treated with lapatinib and capecitabine, indicating that T-DM1 has a favourable safety profile in late-stage patients.^4^

### The TH3RESA study

A separate Phase 3 trial, TH3RESA, compared T-DM1 with the treatment of physician’s choice in patients with advanced breast cancer who had previously been treated with trastuzumab and lapatinib in the advanced setting and a taxane in any setting. Median OS and PFS were both significantly prolonged in the T-DM1-treated group relative to the therapy of physician’s choice, demonstrating that T-DM1 is active in metastatic breast cancer even after multiple lines of prior HER2-directed therapy.^3^

### The MARIANNE study

The MARIANNE study investigated the use of T-DM1 alone or in combination with the HER2 heterodimerisation inhibitor pertuzumab as a first-line treatment for metastatic breast cancer. Although T-DM1 monotherapy and T-DM1 plus pertuzumab did not show statistically superior efficacy compared with trastuzumab plus a taxane, both treatments did show improved tolerability with a reduced frequency of grade 3 or higher adverse events and an increase in health-related quality of life.^5^ Interestingly, there was a trend toward prolonged PFS for T-DM1 over trastuzumab and a taxane in patients previously treated with trastuzumab or lapatinib in neoadjuvant or adjuvant settings, while therapy-naive patients showed no improvement in survival.

### The KRISTINE trial

In the KRISTINE trial, T-DM1 plus pertuzumab was less efficacious than the standard therapy of trastuzumab, pertuzumab, docetaxel and carboplatin in the neoadjuvant setting, providing an inferior rate of pathological complete responses (pCR).^6^ A subgroup of 15 T-DM1 patients who had locoregional progression before surgery showed high heterogeneity of HER2 immunostaining that was associated with low levels of *ERBB2* mRNA and HER2 protein expression, which might have contributed to reduced event-free survival in the T-DM1 treatment arm.^7^ However, the fact that pCR was achieved in 44% of patients who received T-DM1 plus pertuzumab in KRISTINE and the Phase 2 PREDIX trial^8^ without the use of potentially toxic systemic chemotherapy is notable, indicating that a subset of patients, particularly those with high and homogeneous HER expression, might not require neoadjuvant chemotherapy, and that their treatment could be de-escalated.^9^ Other authors have considered why T-DM1 plus pertuzumab does not provide greater efficacy than pertuzumab and trastuzumab in combination with chemotherapy in the metastatic and neoadjuvant settings, proposing tumour heterogeneity, clonal selection, bystander effects and receptor downregulation by competitive binding as potential biological explanations.^10^

### The KATHERINE trial

An interim analysis of the ongoing KATHERINE trial in patients with HER2-positive early breast cancer with residual invasive disease in the breast or axilla after neoadjuvant treatment with trastuzumab and a taxane revealed that use of adjuvant T-DM1 conferred a lower risk of recurrence of invasive breast cancer than adjuvant trastuzumab, although with a higher rate of adverse events.^11^ On the basis of these results, T-DM1 recently received regulatory approval for this neoadjuvant indication.^12^

## Mechanisms of action of T-DM1

**Fig. 2:**
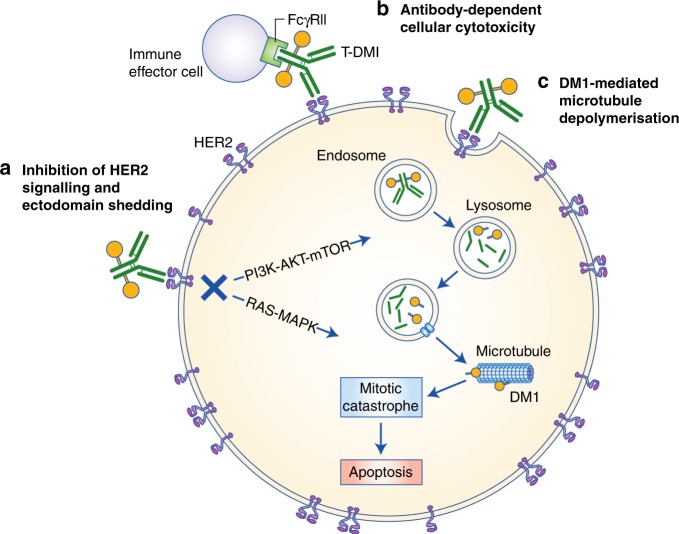
Mechanisms of action of T-DM1. T-DM1 exerts anti-tumour activity via at least three distinct mechanisms. As for trastuzumab, engagement of HER2 receptors by T-DM1 inhibits downstream signalling pathways (via RAS–mitogen-activated protein kinase [MAPK] and phosphatidylinositol 3-kinase [PI3K]–AKT–mammalian target of rapamycin [mTOR]) and ectodomain shedding while also eliciting immune effector cell function (e.g. antibody-dependent cellular cytotoxicity) mediated via Fc receptors. T-DM1–HER2 complexes are also internalised via receptor-mediated endocytosis, after which endocytic vesicles mature through the endosomal pathway for ultimate delivery to lysosomes. Trastuzumab is proteolytically degraded in lysosomes, liberating lysine-MCC-DM1 for active transport into the cytoplasm, where it inhibits tubulin polymerisation resulting in failure of the mitotic spindle and ultimate mitotic catastrophe.

T-DM1 has multiple mechanisms of action, from the selective delivery of DM1 to HER2-positive tumour cells through to trastuzumab-mediated inhibition of HER2 signalling, inhibition of HER2 extracellular domain shedding and induction of antibody-dependent cell-mediated cytotoxicity (ADCC) (Fig. 2).

### Tumour-selective delivery and cytotoxicity of DM1

The tumour selectivity of T-DM1 is conferred by the exquisite specificity of trastuzumab to subdomain IV of the HER2 receptor on the surface of antigen-positive cells. Following binding to this extracellular epitope, the ADC–receptor complex is internalised into early endosomes by receptor-mediated endocytosis. The endocytic vesicles either mature and fuse with the lysosome or are recycled to transport the ADC–receptor complex back to the plasma membrane.^13^ Lysosomal degradation of the antibody component of T-DM1 results in the liberation of lysine-MCC-DM1, which, as a lysine derivative, is positively charged at physiological pH and therefore membrane-impermeable.^14^ As such, lysine-MCC-DM1 requires active transport to efficiently cross the lysosomal membrane before it can engage its molecular target in the cytoplasm and also has a limited ability to diffuse to proximal, antigen-negative cells to induce a cytotoxic bystander effect.^15^ Proteolysis of trastuzumab spares the MCC moiety on DM1, but this retained linker does not impair payload potency, ensuring that lysine-MCC-DM1 liberated from lysosomes binds efficiently to tubulin to prevent microtubule polymerisation.^1,16^ Analogous to vinca alkaloid chemotherapeutics, microtubule depolymerisation by DM1 prevents the assembly of a functional mitotic spindle, resulting in unattached kinetochores, cell-cycle arrest in metaphase with presentation of multinucleated cells and aberrant mitotic figures, and ultimate mitotic catastrophe.^17^

### Trastuzumab-mediated mechanisms of action

In addition to DM1-mediated tubulin poisoning, T-DM1 has also been demonstrated to retain the mechanisms of action of trastuzumab, including blockade of HER2 signalling pathways, inhibition of HER2 ectodomain shedding, and activation of innate and adaptive anti-tumour immunity.^18^ The principal development rationale for trastuzumab was to block the formation of HER2–HER2 homodimers, thereby attenuating ligand-independent activation of downstream phosphatidylinositol 3-kinase (PI3K)–AKT–mammalian target of rapamycin (mTOR) and RAS–mitogen-activated protein kinase (MAPK) signalling pathways and inhibiting cell proliferation, survival, mobility and invasiveness.^19^ Trastuzumab also interferes with the extracellular domain shedding of HER2^20^ that results in the generation of truncated p95HER2 and constitutively activated signalling downstream of the receptor.^21^ ADCC and related immune effects are probably major mechanisms of action for the clinical efficacy of trastuzumab.^22^ The Fc domain of trastuzumab is recognised by cognate Fcγ receptors on natural killer cells, triggering ADCC and the lysis or phagocytosis of trastuzumab-marked HER2-positive tumour cells.^23^ Other proposed, but less clearly validated, mechanisms of action of trastuzumab include inhibition of angiogenesis, increased accumulation of the cyclin-dependent kinase inhibitor p27^Kip1^, and inhibition of DNA repair.^24^

The trastuzumab-dependent actions of T-DM1 might be particularly important in contexts where the intracellular concentrations of lysine-MCC-DM1 are insufficient to directly induce cytotoxicity.^13^ However, in considering their relative contribution to the sum of anti-tumour activity, it is important to consider that the trastuzumab exposures achieved in T-DM1-treated patients are likely to be lower than those in trastuzumab-treated patients, as the clinical dose of T-DM1 is typically lower than that of trastuzumab (3.6 mg/kg q3w compared with 6 mg/kg q3w or 2 mg/kg qw). Consequently, it is plausible—although not explicitly established—that trastuzumab-mediated actions might manifest less strongly in patients treated with T-DM1.

## Mechanisms of resistance to T-DM1

**Fig. 3:**
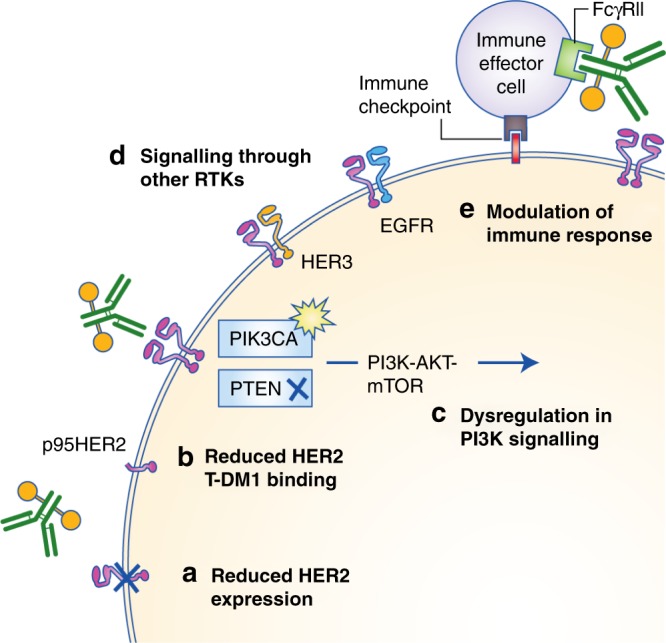
T-DM1 resistance arising from loss of trastuzumab-mediated activity. A reduction in HER2 expression can impair T-DM1 binding and internalisation, preventing the intracellular release of DM1. Shedding of the extracellular domain of HER2 to generate the truncated form, p95HER2, also can prevent T-DM1 binding and DM1 intracellular release. Mutations in *PIK3CA* and loss of function of *PTEN* can lead to constitutive phosphatidylinositol 3-kinase (PI3K)–AKT–mammalian target of rapamycin (mTOR) signalling despite T-DM1-mediated inhibition of HER2. Heterodimerisation of HER2 with HER3 or epidermal growth factor receptor (EGFR) can induce PI3K and mitogen-activated protein kinase (MAPK) signalling in the presence of T-DM1. Finally, immunosuppression can limit the antibody-dependent cell-mediated cytotoxicity (ADCC) associated with T-DM1.

**Fig. 4:**
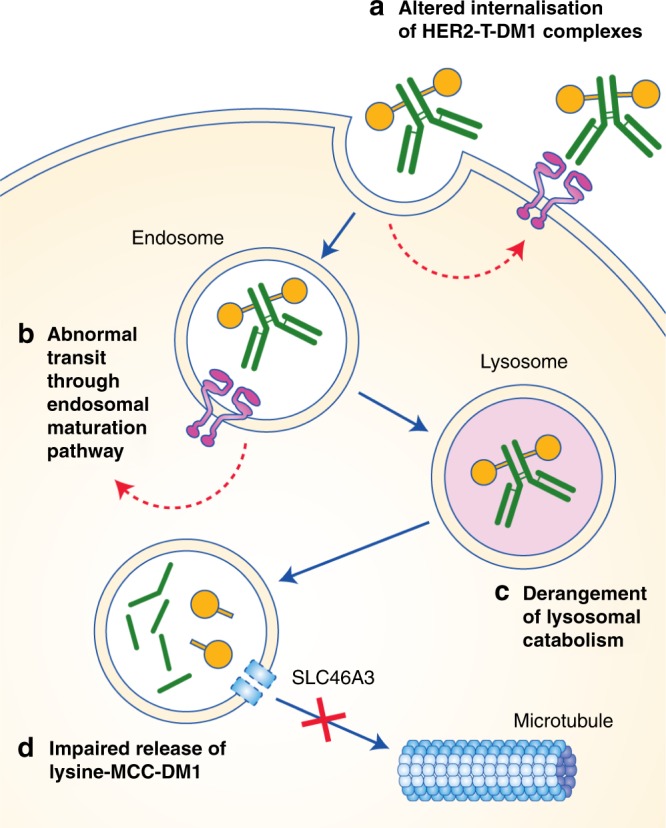
T-DM1 resistance arising from dysfunctional intracellular trafficking and metabolism. HER2–T-DM1 complex internalisation might be reduced by enhanced recycling of HER2–T-DM1 complexes back to the plasma membrane, thereby promoting the efflux of T-DM1. Altered expression of certain endocytic and cytoskeletal proteins could impair normal transit of HER2–T-DM1 complexes through the endosomal maturation pathway. Altered lysosomal pH regulation resulting in decreased acidity of lysosomal vesicles can reduce catabolism of HER2–T-DM1 to lysine-MCC-DM1 and prevent the release of the active compound. Reduced expression of lysosomal transporter proteins, such as SLC46A3, might also impair the release of lysine-MCC-DM1 into the cytoplasm.

**Fig. 5:**
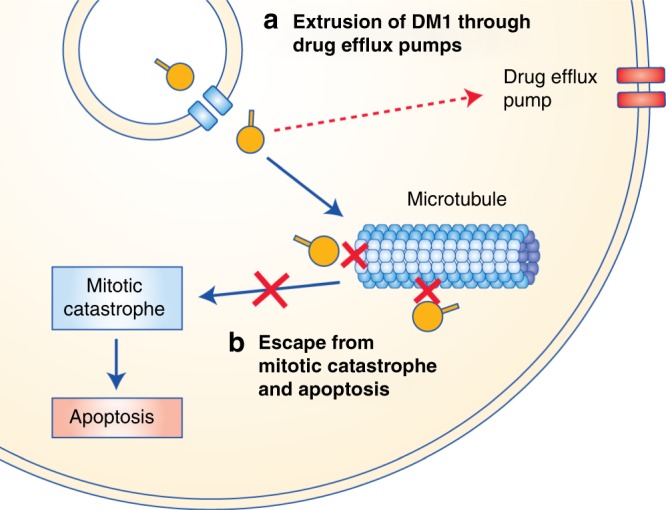
T-DM1 resistance arising from impairment of DM1-mediated cytotoxicity. Increased expression of drug efflux transporters for which DM1 is a substrate might promote the efflux of lysine-MCC-DM1 from cells. Alternatively, cells might escape from DM1-mediated mitotic catastrophe through reduced induction of cyclin B1 or increased expression of polo-like kinase 1 (PLK1), allowing cells to complete mitosis and avoid apoptosis despite having an abnormal mitotic spindle.

As T-DM1 exerts its activity through both trastuzumab and DM1, the observed inherent and acquired resistance to T-DM1 could arise through interference with the action of either or both constituents and, unfortunately, the pharmacological complexity of this agent has confounded efforts to demarcate the clinically important mechanisms. Various potential resistance mechanisms to T-DM1 have been experimentally investigated, relating to the subversion of trastuzumab-mediated effects (Fig. 3), the intracellular trafficking and metabolism of T-DM1 (Fig. 4) and impairment of DM1-mediated cytotoxicity (Fig. 5).

### Subversion of trastuzumab-mediated effects: reduced HER2 expression

A higher expression of HER2 in tumour cells relative to peripheral tissues is a requirement for the safety and efficacy of trastuzumab and T-DM1, and the loss of HER2 is a known resistance mechanism for trastuzumab.^25^ A reduction in HER2 expression would be expected to impair T-DM1 binding and internalisation, limiting not only trastuzumab-mediated anti-tumour activity but also the intracellular release and cytotoxicity of DM1. Decreased HER2 expression relative to parental cells has been seen in a wide range of cell lines selected for T-DM1 resistance by protracted exposure.^26–29^ In a T-DM1-resistant JIMT-1 breast carcinoma cell line, restoring HER2 expression via transfection reversed the resistance phenotype, providing experimental evidence that target expression levels regulate T-DM1 sensitivity.^26^ However, downregulation of HER2 has not been seen in all T-DM1 resistance models, indicating that other mechanisms contribute to treatment failure.^30–32^ Loss of HER2 amplification in tumour cells has been observed in circulating tumour DNA from a small cohort of patients and was found to be associated with T-DM1 resistance in these patients.^33^

### Subversion of trastuzumab-mediated effects: reduced T-DM1 binding

The activity of trastuzumab is dependent on binding to HER2. Truncation of HER2 into p95HER2 can decrease the binding affinity of trastuzumab and limit its activity, although trastuzumab treatment might prevent further truncation.^20^ It is not yet clear whether HER2 truncation can similarly influence T-DM1 activity. The glycoprotein MUC4 can also disrupt trastuzumab binding by interacting directly with HER2,^34^ and *MUC4* knockdown has been shown to increase sensitivity to trastuzumab in JIMT-1 cells.^35^ It is unclear, however, whether an impairment in initial target engagement is also a mechanism of T-DM1 resistance. In one study, T-DM1-resistant NCI-N87 human gastric cells showed ~50% reduced binding of T-DM1 to HER2 relative to parental cells, despite equivalent levels of expression of the receptor in both cell lines^28^; the mechanism underlying reduced HER2 binding was not established. By contrast, in another study, T-DM1–HER2 binding was preserved in all three established T-DM1-resistant cell lines.^27^

### Subversion of trastuzumab-mediated effects: dysregulated PI3K signalling

Activating mutations in *PIK3CA* or loss of function of *PTEN* are prevalent oncogenic events in HER2-positive breast cancer, occurring in 42 and 19% of patients, respectively.^36^ The resulting derangement of PI3K–AKT–mTOR signalling subverts dependence on HER2 dimerisation for maintenance of mitogenic signals and can confer resistance to trastuzumab in preclinical models while being associated with reduced treatment efficacy in some clinical cohorts, particularly in the neoadjuvant setting.^37^ However, it has emerged that PI3K pathway alterations might not be clinically relevant determinants of resistance to T-DM1. Numerically lower rates of pCR were seen in *PIK3CA*-mutant patients treated with neoadjuvant T-DM1 and pertuzumab in the KRISTINE trial, but the *PIK3CA* mutation rate was higher in tumours with low or heterogeneous HER2 expression.^38^ In the EMILIA trial, the presence of *PIK3CA* mutations or loss of *PTEN* was associated with shorter median OS, shorter median PFS and lower overall response rate in patients receiving lapatinib and capecitabine, but had no effect on T-DM1 efficacy.^39^ Similarly, in the TH3RESA trial, T-DM1 conferred benefit regardless of *PIK3CA* mutation status.^40^ A corresponding lack of influence on T-DM1 efficacy has also been observed in *PIK3CA*-mutant and *PTEN*-null preclinical models.^26,27,41^ The apparent lack of impact of PI3K pathway alterations on the activity of T-DM1 presumably reflects the pre-eminence of DM1-mediated (and potentially immunological) effects of this agent and highlights important differences in the resistance mechanisms between trastuzumab and T-DM1.

### Subversion of trastuzumab-mediated effects: signalling through alternative RTKs

Trastuzumab blocks the ligand-independent homodimerisation of HER2 but does not inhibit ligand-induced HER2 heterodimerisation.^42^ Consequently, epidermal growth factor receptor (EGFR)–HER2 and HER2–HER3 heterodimerisation can occur in the presence of trastuzumab to initiate PI3K and MAPK signalling. Indeed, co-expression of HER2 with other HER-family members has been associated with shorter OS in trastuzumab-treated patients, which can be improved with the addition of pertuzumab to inhibit the homodimerisation and heterodimerisation of HER2.^43^ However, there is insufficient evidence to suggest that signalling through other RTKs can reduce the efficacy of T-DM1. Although exogenous administration of the HER3 ligand neuregulin-1β can suppress T-DM1-mediated cytotoxicity in HER2-positive cell lines, and this effect can be reversed by the addition of pertuzumab,^44^ neither HER3 expression,^39,40^ nor the addition of pertuzumab^5,6^ significantly altered the activity of T-DM1 in randomised clinical trials.

Genetic suppression of *EGFR* and treatment with the anti-EGFR antibody cetuximab did not restore sensitivity to T-DM1 in T-DM1-resistant KPL-4 cells despite increased expression of this receptor relative to parental cells, and the addition of the EGFR ligand transforming growth factor α (TGFα) was unable to confer T-DM1 resistance in parental cells.^29^ However, in the EMILIA trial, greater than median EGFR mRNA expression was associated with a shorter median PFS and OS in T-DM1-treated patients.^39^ More research is therefore required to understand the role of EGFR in T-DM1 resistance.

### Subversion of trastuzumab-mediated effects: the tumour immune set point

Clinical data compellingly demonstrate that the immunological status of tumours has a major bearing on the activity of trastuzumab. An inferior response to trastuzumab was seen in HER2-positive breast cancer patients with high levels of infiltrating immunosuppressive regulatory T cells,^45^ high expression of CD73^46^ and expression of the immune checkpoint programmed cell death protein 1 (PD-1) and its ligand PD-L1.^47^ Although analogous clinical data for the response to T-DM1 remain limited, combination therapy with T-DM1 and therapeutic antibodies against both PD-L1 and cytotoxic T-lymphocyte-associated antigen 4 (CTLA-4, another immune checkpoint protein) in the Fo5 HER2-overexpressing transgenic mouse model markedly increased efficacy relative to monotherapy. Combination therapy resulted in cures in nearly 100% of animals, with those free of disease immune to tumour re-challenge, indicating the occurrence of treatment-induced T-cell memory.^48^ Interestingly, immune-mediated cytotoxicity has been seen in a trastuzumab-resistant tumour model in response to T-DM1 but not to trastuzumab, suggesting the action of DM1 might independently trigger immune activation.^48^ Understanding the role of the immune set point in determining the activity of T-DM1 is a key priority. In a Phase 2 study, the addition of the PD-L1 inhibitor atezolizumab to T-DM1 induced a numerical increase in median PFS in previously treated PD-L1-positive HER2-positive advanced breast cancer patients,^49^ while a Phase 1b study to evaluate the safety, tolerability and activity of the PD-1 inhibitor pembrolizumab with T-DM1 in HER2-positive metastatic breast cancer is underway (Trial ID: NCT03032107).

### Intra-tumour heterogeneity in HER2 expression and accessibility

The impact of intra-tumour heterogeneity in HER2 expression on the efficacy of T-DM1 without concurrent chemotherapy was alluded to in our discussion of the KRISTINE trial. This phenomenon was investigated prospectively in a single-arm Phase 2 neoadjuvant study of T-DM1 plus pertuzumab.^50^ Among the ten (out of 157 evaluable) patients in this trial determined to exhibit intra-tumour HER2 heterogeneity (defined as either HER2 positivity by FISH in 5–50% of tumour cells in at least one of six cores per tumour, or presence of a HER2-negative tumour area), none showed pathological complete responses (pCR), meeting the primary endpoint for this association. Target heterogeneity was also explored in the ZEPHIR trial, which utilised ^89^Zr-trastuzumab PET to assess intra-/inter-patient heterogeneity in HER2 expression in the metastatic setting.^51^ Of the 56 patients assessed, 46% showed ^89^Zr-trastuzumab avidity in less than half of their FDG-PET/CT tumour volume and experienced shorter time to treatment failure.

### Altered internalisation of HER2–T-DM1 complexes

Once T-DM1 has engaged the HER2 receptor, the complex requires internalisation into endosomes prior to transitioning to lysosomes. However, increased recycling of HER2-containing endosomes back to the plasma membrane might divert T-DM1 from trafficking to the lysosome and thus attenuate its DM1-mediated anti-tumour activity. Rapid recycling is a feature of HER2 and has been observed upon trastuzumab binding.^52^ Moreover, enhanced recycling of HER2–T-DM1 complexes has been observed in T-DM1-resistant JIMT-1 cells and was associated with reduced DM1 intracellular release.^26^ However, overall levels of HER2 protein were lower in the resistant cells, which might also have contributed to the resistance phenotype. In other T-DM1-resistant cell lines, minimal changes in the magnitude and rate of HER2–T-DM1 internalisation have been observed, demonstrating incomplete penetrance of this potential resistance mechanism.^27,30,31^

Internalisation and degradation of HER2–trastuzumab complexes, as opposed to recycling back to the membrane, can be promoted by the endocytic scaffolding protein endophilin A2 (encoded by *SH3GL1*),^41^ and would be expected to generate intracellular release of DM1 and consequently induction of cell death. Knockdown of *SH3GL1* in HER2-positive HCC1954 and SK-BR-3 breast cancer cells reduced HER2 internalisation and markedly suppressed T-DM1-mediated cytotoxicity.^41^ Endophilin A2 might, therefore, provide an effective marker of intrinsic sensitivity to T-DM1 given its potential role in T-DM1 internalisation and the association of *SH3GL1* expression with poor relapse-free survival and OS in patients with node-positive, HER2-positive breast cancer;^41^ however, further investigation in additional experimental models is required.

### Abnormal transit of HER2–T-DM1 through the endosomal maturation pathway

The cellular machinery that mediates the internalisation of HER2–T-DM1 complexes remains poorly understood. One possible mechanism of resistance identified by Sung et al.,^28^ might be conferred by caveolin-mediated endocytosis, which could facilitate internalisation of T-DM1 but limit its subsequent journey into lysosomes. In this in vitro study, proteins involved in caveolae formation and endocytosis, caveolin-1 and cavin-1, showed elevated expression in T-DM1-resistant NCI-N87 cells, with increased co-localisation of caveolin-1 and T-DM1 and decreased lysosomal sequestration of T-DM1 in resistant cells relative to parental cells. However, the role of caveolin-mediated endocytosis in T-DM1 resistance is contentious as, in the same study, shRNA-mediated knockdown of caveolin-1 (*CAV1*) was insufficient to re-sensitise resistant NCI-N87 cells to T-DM1.^28^ Furthermore, Chung et al.^53,54^ reported opposing findings: overexpression of *CAV1* in BT-474 breast cancer cells or upregulation through pre-treatment with trastuzumab or metformin increased T-DM1 sensitivity, while siRNA-mediated *CAV1* knockdown in HER2-positive SK-BR-3 cells reduced sensitivity. Further research is required to confirm the relevance of the role of caveolin-mediated endocytosis in the clinical resistance of T-DM1.

Elevated expression of additional proteins involved in vesicle transport or cytoskeletal remodelling, such as RAB GTPase-activating protein 1 (RABGAP1), p21-activated kinase 4 (PAK4) and HECT domain E3 ubiquitin protein ligase 1 (HECTD1), has been reported in T-DM1-resistant JIMT-1 cells,^26^ although it is unclear whether these changes are a cause or consequence of drug resistance. Altered cell adhesion and prostaglandin signalling have also been observed in T-DM1-resistant oesophageal carcinoma cells.^55^ These changes might promote migration, survival or MAPK signalling, which could affect T-DM1 transit or the response of the cell to microtubule depolymerisation, but the translatability of findings in oesophageal cancer models to HER2-positive breast cancer might be limited.

### Derangement of lysosomal catabolism of HER2–T-DM1 complexes

Lysosomes are specialised acidic compartments, and their catabolic capacity relies on the vacuolar H^+^-ATPase (V-ATPase) proton pump for pH regulation. As T-DM1 requires lysosomal proteolysis, it is notable that T-DM1-resistant NCI-N87 gastric carcinoma cells showed diminished lysosome acidification and reduced intracellular concentrations of lysine-MCC-DM1 relative to parental cells.^31^ Moreover, small-molecule inhibition of V-ATPase antagonised the activity of T-DM1 in wild-type NCI-N87 cells but not in resistant NCI-N87 cells, suggesting that aberrant V-ATPase activity can decrease T-DM1 metabolism and promote resistance. Similarly, T-DM1-resistant BT-474 clones showed an accumulation of T-DM1 in lysosomes with elevated pH, as well as decreased activity of cathepsin B, a pH-dependent lysosomal proteolytic enzyme.^32^ As with NCI-N87 cells, raising lysosomal pH in wild-type BT-474 cells by V-ATPase inhibition reduced their sensitivity to T-DM1, demonstrating that lysosomal function is crucial in determining intrinsic cellular sensitivity to T-DM1.

### Impaired lysosomal release of lysine-MCC-DM1

Due to its positive charge, lysine-MCC-DM1 is unable to passively diffuse across the lysosomal membrane and therefore requires active transport before it can inhibit tubulin polymerisation in the cytoplasm.^14^ In a functional genomic screen of 2601 candidate transporter genes, *SLC46A3* was identified to encode a lysosomal membrane protein capable of transporting maytansine-based linker-drug catabolites.^56^ Its knockdown reduced the potency of T-DM1 and other DM1-armed ADCs regardless of linker chemistry but did not affect alternative payloads, indicating that DM1 itself, rather than the linker or lysine component, is likely to be a substrate for SLC46A3. Two independent studies reported loss of *SLC46A3* expression in T-DM1-resistant BT-474 and NCI-N87 cells, in which sensitivity to the agent could be restored by forced expression of *SLC46A3*, and in a T-DM1-resistant HER2-positive invasive ductal carcinoma patient-derived xenograft model.^29,57^ Functionally, knockdown of *SLC46A3* in BT-474 or knockout in SK-BR-3 cells markedly reduced sensitivity to T-DM1. SLC46A3 expression, which is high in breast cancer cell lines but varies between other tumour types, might, therefore, be a predictor of T-DM1 sensitivity and has been proposed as a potential patient selection biomarker, with patients or cancer types (e.g. multiple myeloma) that express low levels of SLC46A3, and therefore potentially less sensitive to T-DM1, being more suited to alternative payloads that do not require SLC46A3-mediated transport.^57^

### Impairment of DM1-mediated cytotoxicity: increased expression of drug efflux transporters

Since DM1 and other maytansinoids are substrates for multidrug-resistance transporters,^58^ the upregulation of these transporters could promote the efflux of DM1 from cells and confer cellular resistance to T-DM1. Upregulation of *ABCC1* (which encodes multidrug-resistance-associated protein 1 [MRP1]), *ABCC2* (MRP2), *ABCC4* (MRP4), *ABCB1* (multidrug-resistance protein 1 [MDR1, P-glycoprotein)] and *ABCG2* (breast cancer resistance protein [BCRP]), promoting DM1 efflux, has been reported in T-DM1-resistant cell lines.^26,29,59^ In these studies, pharmacological inhibition of MRP1, MRP2 and MDR1 reversed T-DM1 resistance, whereas BCRP inhibition and *ABCC4* knockdown did not. Separately, inhibition of MDR1 but not MRP1 or BCRP enhanced the potency of an MCC-DM1 ADC, while extrusion by MDR1 was reduced by altering the linker group, which prevented MDR1-mediated resistance in MDR1-expressing cells and xenografts.^58^ However, other studies have reported minimal changes in the expression of drug efflux transporters in T-DM1-resistant cells,^32,55^ suggesting that lysine-MCC-DM1 extrusion is unlikely to be a major mechanism of resistance in all cases.

### Impairment of DM1-mediated cytotoxicity: escape from mitotic catastrophe and apoptosis

The cytotoxicity of DM1 depends on the cell attempting to undergo mitosis in the context of incomplete spindle formation, resulting in mitotic catastrophe and apoptosis.^13^ Cells that avoid this process might gain resistance to T-DM1. One manner in which cells have been reported to avert T-DM1-induced mitotic catastrophe is through the reduced induction of cyclin B1, a protein that is essential for the activation of cyclin-dependent kinase 1 (CDK1) and progression into M-phase, with degradation required for exit from mitosis.^60^ This mechanism of resistance to mitosis-targeting agents has been termed ‘mitotic slippage’. Despite a defective mitotic spindle, reduced induction of cyclin B1/CDK1 results in an inability to halt cell-cycle progression, consequently leading to mitotic exit and cytokinesis, and thus avoidance of mitotic catastrophe. This scenario promotes the accumulation of genetic instability and a more malignant phenotype, as daughter cells inherit karyotypic atypia due to improper chromosome segregation at anaphase.^61^

In two separate studies, T-DM1 treatment increased the fraction of HCC1419, HCC1954, SK-BR-3 and BT-474 cells that became arrested in the G_2_/M phase through elevated cyclin B1 expression, but in resistant clones of the same cell lines, no increase in cyclin B1 expression or CDK1 activity was observed, with minimal G_2_/M phase arrest and attenuation of mitotic catastrophe and apoptosis.^27,30^ Knockdown of *CDC20* to promote cyclin B1 expression increased the sensitivity of two T-DM1 resistant cell lines to T-DM1, whereas knockdown of cyclin B1 (*CCNB1*) in parental cells induced T-DM1 resistance.^27^ Expression of the mitotic kinase polo-like kinase 1 (PLK1) has been reported to be significantly increased in T-DM1-resistant SK-BR-3 and BT-474 cells compared with their wild-type counterparts and has been hypothesised to play a role in the completion of mitosis despite the presence of an abnormal mitotic spindle by preventing CDK1 activation.^30^ Inhibition of PLK1 with volasertib reversed T-DM1 resistance, inducing growth inhibition with mitotic arrest and DNA damage profiles equivalent to those reported in T-DM1-sensitive cells.^30^

## Strategies for overcoming T-DM1 resistance

Resistance to T-DM1 remains a major clinical challenge, and the results of preclinical studies described in this review indicate strategies that could be explored to overcome resistance. A synthesis of the current literature supports the view that resistance mechanisms with the strongest evidence base relate to derangement of intracellular transport and metabolism of T-DM1 and to attenuation of lysine-MCC-DM1-mediated cytotoxicity, although evidence for mechanisms related to internalisation, abnormal transit, lysosomal catabolism and drug efflux have been observed in a limited number of experimental models that were not patient-derived. Mechanisms relating to insensitivity to HER2 signalling blockade are less strongly supported, although the role of EGFR and immunological phenomena require further investigation. Accordingly, approaches to circumvent resistance that are strongly aligned with the experimental evidence include the use of alternative linker-payload chemistry to generate more effective second-generation ADCs (e.g. trastuzumab deruxtecan, trastuzumab duocarmazine) that can also obviate the T-DM1 resistance mechanisms that appear specific to lysine-MCC-DM1, and the use of combination therapies that target the molecular circuitry implicated in the loss of DM1 sensitivity.

### Trastuzumab deruxtecan (DS-8201a)

Trastuzumab deruxtecan (DS-8201a; Daiichi Sankyo) is an ADC that comprises a humanised monoclonal IgG1 anti-HER2 antibody produced using an identical amino acid sequence to trastuzumab, linked by a self-immolative maleimide linker to the topoisomerase I inhibitor, DXd, a derivative of exatecan.^62^ The DAR for trastuzumab deruxtecan is much higher than for T-DM1 (7.7 versus 3.5), allowing for greater payload delivery per antigen engagement event while avoiding rapid in vivo clearance, which can occur with high DAR and result in off-target toxicities through increased exposure to the payload in plasma.^62^ Trastuzumab deruxtecan has shown activity in T-DM1-resistant cell lines and patient-derived xenograft models and has been proposed to be a suitable treatment option for tumours with low HER2 expression that are less sensitive to T-DM1.^59,62^ The agent is currently in Phase 2 and 3 trials for the treatment of HER2-positive metastatic breast cancer, gastric, colorectal, uterine and non-small cell lung cancers plus carcinosarcoma, following evidence of anti-tumour activity from Phase 1 data, in which 81 out of 160 evaluated patients showed durable RECIST-confirmed responses in HER2-positive metastatic breast cancer treated with prior T-DM1, HER2-positive gastric cancer treated with prior trastuzumab, HER2-low metastatic breast cancer and other HER2-expressing solid tumours^63–66^

Several plausible explanations could account for the apparent activity of trastuzumab deruxtecan in tumours that progressed on prior T-DM1. First, owing to the differences in linker-payload chemistry, trastuzumab deruxtecan is unlikely to be affected by resistance mechanisms that are specific to DM1.^26^ Secondly, the DXd payload might not be extruded as efficiently by efflux transporters as DM1.^59^ Thirdly, trastuzumab deruxtecan, but not T-DM1, has shown a bystander effect, whereby neighbouring HER2-negative cells incurred cytotoxicity in vitro and in vivo, most probably due to the enhanced membrane permeability and thus transcellular diffusion of the DXd payload relative to DM1.^15^ This bystander effect might be significant in suppressing the outgrowth of some resistant clones by no longer requiring target expression and intracellular payload release within each cell for cytotoxicity.

### Trastuzumab duocarmazine (SYD985)

[vic-]trastuzumab duocarmazine (SYD985, Synthon), another HER2-targeting ADC, comprises a valine-citrulline-*seco* duocarmycin hydroxybenzamide azaindole (vc-*seco*-DUBA) payload conjugated to trastuzumab by a cleavable linker at a DAR of 2.8.^67^ vc-*seco*-DUBA is a potent duocarmycin analogue that binds to the minor groove of DNA causing irreversible DNA alkylation and cell death. Preclinically, SYD985 is active against a range of HER2-expressing cell lines and tumour models, including breast, ovarian and uterine carcinomas. Like trastuzumab deruxtecan, SYD985 is also active in cell lines and tumour models expressing low levels of HER2, potentially owing to the bystander effect of its payload, where degradation of the cleavable linker in tumour cells by proteases such as cathepsin B releases the membrane-penetrant payload to passively diffuse to proximal antigen-negative or antigen-low cells.^68,69^ SYD985 has shown promising efficacy in Phase 1 trials in heavily pre-treated patients with HER2-positive, T-DM1-resistant and HER2-low metastatic breast cancer^70^ and is currently undergoing Phase 3 evaluation for HER2-positive, unresectable, locally-advanced or metastatic breast cancer.

### Combination therapies to limit T-DM1 resistance

Although second-generation HER2-targeting ADCs are showing promising early-phase clinical activity, many of the T-DM1 resistance mechanisms that limit the action of trastuzumab or Fc-mediated ADCC are likely to apply to these agents, and additional resistance mechanisms relevant to their respective linker-payloads might well arise in advanced disease. As such, the extent to which the clinical activity of these second-generation HER2-targeting ADCs will be limited by drug resistance remains to be seen. An alternative approach to overcoming T-DM1 resistance is to use this drug in combination with agents that perturb the cell biological pathways implicated in T-DM1 resistance, an approach that might be feasible given the wide therapeutic index of T-DM1. Multiple agents that target canonical trastuzumab-resistance mechanisms, such as signalling through alternative RTKs (e.g. neratinib, pertuzumab), PI3K pathway activation (e.g. alpelisib), HER2 truncation (e.g. tucatinib) and immune checkpoints (e.g. pembrolizumab, atezolizumab) have been tested clinically in combination with T-DM1, as has the CDK4/6 inhibitor ribociclib for co-inhibition of targets downstream of the HER2 pathway,^71^ with some studies showing promising activity.^72,73^ Limiting DM1-mediated resistance could potentially also be achieved by combination therapy with small-molecule inhibitors of multidrug-resistance transporters; however, these inhibitors have seen limited clinical application due to the wide role of efflux transporters in xenobiotic elimination and their abundant expression in non-tumour tissue.^74^ Another potential approach is to pharmacologically target mechanisms that promote escape from mitotic catastrophe, as demonstrated by the ability of the combination of T-DM1 and the PLK1 inhibitor volasertib to overcome T-DM1 resistance by inducing mitotic arrest in vitro,^30^ which warrants further research.

## Conclusions

T-DM1 is a safe and effective therapy for HER2-positive breast cancer, but its clinical activity is limited by intrinsic and acquired resistance. Most evidence points to altered trafficking/metabolism of T-DM1 and impairment of lysine-MCC-DM-1-mediated cytotoxicity being the predominant mechanisms of T-DM1 resistance, but subversion of trastuzumab-mediated effects, particularly ADCC and HER2/EGFR expression, is also implicated. Despite various mechanisms of resistance having been proposed, further understanding is required to develop therapeutic strategies to overcome this resistance and to assess whether any of the resistance mechanisms could provide biomarkers for patient stratification for T-DM1 therapy. Alternative therapeutic approaches include the use of second-generation HER2-targeting ADCs that utilise distinct payloads and cleavable linkers that mediate activity in HER2-low cells, as well as therapeutic combinations to overcome trastuzumab-mediated or DM1-mediated resistance. These strategies are already showing promise in clinical trials and have the potential to provide further survival improvements for patients with HER2-positive cancer.

## Data Availability

Not applicable.

## References

[1] Lewis Phillips GD, Li G, Dugger DL, Crocker LM, Parsons KL, Mai E (2008). Targeting HER2-positive breast cancer with trastuzumab-DM1, an antibody-cytotoxic drug conjugate. Cancer Res..

[2] Verma S, Miles D, Gianni L, Krop IE, Welslau M, Baselga J (2012). Trastuzumab emtansine for HER2-positive advanced breast cancer. N. Engl. J. Med..

[3] Krop IE, Kim SB, Martin AG, LoRusso PM, Ferrero JM, Badovinac-Crnjevic T (2017). Trastuzumab emtansine versus treatment of physician’s choice in patients with previously treated HER2-positive metastatic breast cancer (TH3RESA): final overall survival results from a randomised open-label phase 3 trial. Lancet Oncol..

[4] Welslau M, Diéras V, Sohn JH, Hurvitz SA, Lalla D, Fang L (2014). Patient-reported outcomes from EMILIA, a randomized phase 3 study of trastuzumab emtansine (T-DM1) versus capecitabine and lapatinib in human epidermal growth factor receptor 2-positive locally advanced or metastatic breast cancer. Cancer.

[5] Perez EA, Barrios C, Eiermann W, Toi M, Im YH, Conte P (2017). Trastuzumab emtansine with or without pertuzumab versus trastuzumab plus taxane for human epidermal growth factor receptor 2-positive, advanced breast cancer: Primary results from the phase III MARIANNE study. J. Clin. Oncol..

[6] Hurvitz SA, Martin M, Symmans WF, Jung KH, Huang CS, Thompson AM (2018). Neoadjuvant trastuzumab, pertuzumab, and chemotherapy versus trastuzumab emtansine plus pertuzumab in patients with HER2-positive breast cancer (KRISTINE): a randomised, open-label, multicentre, phase 3 trial. Lancet Oncol..

[7] Hurvitz SA, Martin M, Jung KH, Huang C-S, Harbeck N, Valero V (2019). Neoadjuvant trastuzumab emtansine and pertuzumab in human epidermal growth factor receptor 2–positive breast cancer: Three-year outcomes from the phase III KRISTINE study. J. Clin. Oncol..

[8] Bergh JCS, Andersson A, Bjohle J, Bosch A, Carlsson L, Dreifaldt AC (2019). Docetaxel, trastuzumab, pertuzumab versus trastuzumab emtansine as neoadjuvant treatment of HER2-positive breast cancer: Results from the Swedish PREDIX HER2 trial identifying a new potential de-escalation standard?. J. Clin. Oncol..

[9] Okines A (2017). TDM1 in the neoadjuvant treatment of HER2 positive breast cancer: Impact of the KRISTINE (TRIO-021) trial. Rev. Recent Clin. Trials.

[10] Ocaña A, Amir E, Pandiella A (2018). Dual targeting of HER2-positive breast cancer with trastuzumab emtansine and pertuzumab: Understanding clinical trial results. Oncotarget.

[11] von Minckwitz G, Huang C-S, Mano MS, Loibl S, Mamounas EP, Untch M (2019). Trastuzumab emtansine for residual invasive HER2-positive breast cancer. N. Engl. J. Med..

[12] FDA approves ado-trastuzumab emtansine for early breast cancer. 2019. https://www.fda.gov/drugs/resources-information-approved-drugs/fda-approves-ado-trastuzumab-emtansine-early-breast-cancer.

[13] Barok M, Joensuu H, Isola J (2014). Trastuzumab emtansine: mechanisms of action and drug resistance. Breast Cancer Res..

[14] Erickson HK, Park PU, Widdison WC, Kovtun YV, Garrett LM, Hoffman K (2006). Antibody-maytansinoid conjugates are activated in targeted cancer cells by lysosomal degradation and linker-dependent intracellular processing. Cancer Res..

[15] Ogitani Y, Hagihara K, Oitate M, Naito H, Agatsuma T (2016). Bystander killing effect of DS-8201a, a novel anti-human epidermal growth factor receptor 2 antibody-drug conjugate, in tumors with human epidermal growth factor receptor 2 heterogeneity. Cancer Sci..

[16] Issell BF, Crooke ST (1978). Maytansine. Cancer Treat. Rev..

[17] Barok M, Tanner M, Köninki K, Isola J (2011). Trastuzumab-DM1 is highly effective in preclinical models of HER2-positive gastric cancer. Cancer Lett..

[18] Junttila TT, Li G, Parsons K, Phillips GL, Sliwkowski MX (2011). Trastuzumab-DM1 (T-DM1) retains all the mechanisms of action of trastuzumab and efficiently inhibits growth of lapatinib insensitive breast cancer. Breast Cancer Res. Treat.

[19] Hudis CA (2007). Trastuzumab—mechanism of action and use in clinical practice. N. Engl. J. Med..

[20] Molina MA, Codony-Servat J, Albanell J, Rojo F, Arribas J (2001). Baselga J. Trastuzumab (Herceptin), a humanized anti-HER2 receptor monoclonal antibody, inhibits basal and activated HER2 ectodomain cleavage in breast cancer cells. Cancer Res..

[21] Scaltriti M, Rojo F, Ocaña A, Anido J, Guzman M, Cortes J (2007). Expression of p95HER2, a truncated form of the HER2 receptor, and response to Anti-HER2 therapies in breast cancer. J. Natl Cancer Inst..

[22] Gennari R, Menard S, Fagnoni F, Ponchio L, Scelsi M, Tagliabue E (2004). Pilot study of the mechanism of action of preoperative trastuzumab in patients with primary operable breast tumors overexpressing HER2. Clin. Cancer Res..

[23] Cooley S, Burns LJ, Repka T, Miller JS (1999). Natural killer cell cytotoxicity of breast cancer targets is enhanced by two distinct mechanisms of antibody-dependent cellular cytotoxicity against LFA-3 and HER2/neu. Exp. Hematol..

[24] Spector NL, Blackwell KL (2009). Understanding the mechanisms behind trastuzumab therapy for human epidermal growth factor receptor 2-positive breast cancer. J. Clin. Oncol..

[25] Mittendorf EA, Wu Y, Scaltriti M, Meric-Bernstam F, Hunt KK, Dawood S (2009). Loss of HER2 amplification following trastuzumab-based neoadjuvant systemic therapy and survival outcomes. Clin. Cancer Res..

[26] Loganzo F, Tan X, Sung M, Jin G, Myers JS, Melamud E (2015). Tumor cells chronically treated with a trastuzumab-maytansinoid antibody-drug conjugate develop varied resistance mechanisms but respond to alternate treatments. Mol. Cancer Ther..

[27] Sabbaghi MA, Gil-Gomez G, Guardia C, Servitja S, Arpí O, García-Alonso S (2017). Defective cyclin B1 induction in trastuzumab-emtansine (T-DM1) acquired resistance in HER2-positive breast cancer. Clin. Cancer Res..

[28] Sung M, Tan X, Lu B, Golas J, Hosselet C, Wang F (2017). Caveolae-mediated endocytosis as a novel mechanism of resistance to trastuzumab emtansine (T-DM1). Mol. Cancer Ther..

[29] Li G, Guo J, Shen B-Q, Yadav DB, Sliwkowski MX, Crocker LM (2018). Mechanisms of acquired resistance to trastuzumab emtansine in breast cancer cells. Mol. Cancer Ther..

[30] Saatci Ö, Borgoni S, Akbulut Ö, Durmuş S, Raza U, Eyüpoğlu E (2018). Targeting PLK1 overcomes T-DM1 resistance via CDK1-dependent phosphorylation and inactivation of Bcl-2/xL in HER2-positive breast cancer. Oncogene.

[31] Wang H, Wang W, Xu Y, Yang Y, Chen X, Quan H (2017). Aberrant intracellular metabolism of T-DM1 confers T-DM1 resistance in human epidermal growth factor receptor 2-positive gastric cancer cells. Cancer Sci..

[32] Ríos-Luci C, García-Alonso S, Díaz-Rodríguez E, Nadal-Serrano M, Arribas J, Ocaña A (2017). Resistance to the antibody–drug conjugate T-DM1 is based in a reduction in lysosomal proteolytic activity. Cancer Res..

[33] Sakai H, Tsurutani J, Iwasa T, Komoike Y, Sakai K, Nishio K (2018). HER2 genomic amplification in circulating tumor DNA and estrogen receptor positivity predict primary resistance to trastuzumab emtansine (T-DM1) in patients with HER2-positive metastatic breast cancer. Breast Cancer.

[34] Price-Schiavi SA, Jepson S, Li P, Arango M, Rudland PS, Yee L (2002). Rat MUC4 (sialomucin complex) reduces binding of anti-ErbB2 antibodies to tumor cell surfaces, a potential mechanism for herceptin resistance. Int. J. Cancer.

[35] Nagy P, Friedländer E, Tanner M, Kapanen AI, Carraway KL, Isola J (2005). Decreased accessibility and lack of activation of ErbB2 in JIMT-1, a Herceptin-resistant, MUC4-expressing breast cancer cell line. Cancer Res..

[36] The Cancer Genome Atlas Network. (2012). Comprehensive molecular portraits of human breast tumours. Nature.

[37] Loibl S, Majewski I, Guarneri V, Nekljudova V, Holmes E, Bria E (2016). PIK3CA mutations are associated with reduced pathological complete response rates in primary HER2-positive breast cancer: pooled analysis of 967 patients from five prospective trials investigating lapatinib and trastuzumab. Ann. Oncol..

[38] de Haas S, Hurvitz S, Martin M, Kiermaier A, Lewis Phillips G, Xu J (2017). Biomarker analysis from the neoadjuvant KRISTINE study in HER2-positive early breast cancer (EBC). Cancer Res..

[39] Baselga J, Phillips GDL, Verma S, Ro J, Huober J, Guardino AE (2016). Relationship between tumor biomarkers and efficacy in EMILIA, a phase III study of trastuzumab emtansine in HER2-positive metastatic breast cancer. Clin. Cancer Res..

[40] Kim SB, Wildiers H, Krop IE, Smitt M, Yu R, Lysbet de Haas S (2016). Relationship between tumor biomarkers and efficacy in TH3RESA, a phase III study of trastuzumab emtansine (T-DM1) vs. treatment of physician’s choice in previously treated HER2-positive advanced breast cancer. Int. J. Cancer.

[41] Baldassarre T, Truesdell P, Craig AW (2017). Endophilin A2 promotes HER2 internalization and sensitivity to trastuzumab-based therapy in HER2-positive breast cancers. Breast Cancer Res..

[42] Ghosh R, Narasanna A, Wang SE, Liu S, Chakrabarty A, Balko JM (2011). Trastuzumab has preferential activity against breast cancers driven by HER2 homodimers. Cancer Res..

[43] Luque-Cabal M, García-Teijido P, Fernández-Pérez Y, Sánchez-Lorenzo L, Palacio-Vázquez I (2016). Mechanisms behind the resistance to trastuzumab in HER2-amplified breast cancer and strategies to overcome it. Clin. Med. Insights Oncol..

[44] Phillips GDL, Fields CT, Li G, Dowbenko D, Schaefer G, Miller K (2014). Dual targeting of HER2-positive cancer with trastuzumab emtansine and pertuzumab: Critical role for neuregulin blockade in antitumor response to combination therapy. Clin. Cancer Res..

[45] Force J, Howie LJ, Abbott SE, Bentley R, Marcom PK, Kimmick G (2018). Early stage HER2-positive breast cancers not achieving a pCR from neoadjuvant rrastuzumab- or pertuzumab-based regimens have an immunosuppressive phenotype. Clin. Breast Cancer.

[46] Turcotte M, Allard D, Mittal D, Bareche Y, Buisseret L, Jose V (2017). CD73 promotes resistance to HER2/ErbB2 antibody therapy. Cancer Res..

[47] Gianni L, Bianchini G, Valagussa P, Belousov A, Thomas M, Pusztai L (2012). Adaptive immune system and immune checkpoints are associated with response to pertuzumab (P) and trastuzumab (H) in the NeoSphere study. Cancer Res..

[48] Müller, P., Kreuzaler, M., Khan, T., Thommen, D. S., Martin, K., Glatz, K. et al. Trastuzumab emtansine (T-DM1) renders HER2 + breast cancer highly susceptible to CTLA-4/PD-1 blockade. *Sci. Transl. Med*. **7**, pp. 315ra188 (2015). 10.1126/scitranslmed.aac4925 26606967

[49] Emens L, Esteva F, Beresford M, Saura C, De Laurentiis M, Kim S-B (2019). Results from KATE2, a randomized phase 2 study of atezolizumab (atezo)+trastuzumab emtansine (T-DM1) vs placebo (pbo)+T-DM1 in previously treated HER2+ advanced breast cancer (BC). Cancer Res..

[50] Filho OM, Viale G, Trippa L, Li T, Yardley DA, Mayer IA (2019). HER2 heterogeneity as a predictor of response to neoadjuvant T-DM1 plus pertuzumab: Results from a prospective clinical trial. J. Clin. Oncol..

[51] Gebhart G, Lamberts LE, Wimana Z, Garcia C, Emonts P, Ameye L (2016). Molecular imaging as a tool to investigate heterogeneity of advanced HER2-positive breast cancer and to predict patient outcome under trastuzumab emtansine (T-DM1): The ZEPHIR trial. Ann. Oncol..

[52] Austin CD, De Mazière AM, Pisacane PI, Van Dijk SM, Eigenbrot C, Sliwkowski MX (2004). Endocytosis and sorting of ErbB2 and the site of action of cancer therapeutics trastuzumab and geldanamycin. Mol. Biol. Cell..

[53] Chung, Y. C., Kuo, J. F., Wei, W. C., Chang, K. J., Chao, W. T., Tan, M. Caveolin-1 dependent endocytosis enhances the chemosensitivity of HER-2 positive breast cancer cells to trastuzumab emtansine (T-DM1). *PLoS ONE* **10**, e0133072 10.1371/journal.pone.0133072 (2015). 10.1371/journal.pone.0133072 PMC4501549 26172389

[54] Chung, Y. C., Chang, C. M., Wei, W. C., Chang, T. W., Chang, K. J., Chao, W. T. Metformin-induced caveolin-1 expression promotes T-DM1 drug efficacy in breast cancer cells. *Sci. Rep.* **8**, 3930 10.1038/s41598-018-22250-8 (2018). 10.1038/s41598-018-22250-8 PMC5834501 29500444

[55] Sauveur J, Matera E-L, Chettab K, Valet P, Guitton J, Savina A (2018). Esophageal cancer cells resistant to T-DM1 display alterations in cell adhesion and the prostaglandin pathway. Oncotarget.

[56] Hamblett KJ, Jacob AP, Gurgel JL, Tometsko ME, Rock BM, Patel SK (2015). SLC46A3 is required to transport catabolites of noncleavable antibody maytansine conjugates from the lysosome to the cytoplasm. Cancer Res..

[57] Kinneer K, Meekin J, Tiberghien AC, Tai Y-T, Phipps S, Kiefer CM (2018). SLC46A3 as a potential predictive biomarker for antibody–drug conjugates bearing noncleavable linked maytansinoid and pyrrolobenzodiazepine warheads. Clin. Cancer Res..

[58] Kovtun YV, Audette CA, Mayo MF, Jones GE, Doherty H, Maloney EK (2010). Antibody-maytansinoid conjugates designed to bypass multidrug resistance. Cancer Res..

[59] Takegawa N, Nonagase Y, Yonesaka K, Sakai K, Maenishi O, Ogitani Y (2017). DS-8201a, a new HER2-targeting antibody-drug conjugate incorporating a novel DNA topoisomerase I inhibitor, overcomes HER2-positive gastric cancer T-DM1 resistance. Int. J. Cancer.

[60] Gavet O, Pines J (2010). Progressive activation of CyclinB1-Cdk1 coordinates entry to mitosis. Dev. Cell.

[61] Visconti, R., Della Monica, R., Grieco, D. Cell cycle checkpoint in cancer: a therapeutically targetable double-edged sword. *J. Exp. Clin. Cancer Res*. **35**, 153 10.1186/s13046-016-0433-9 (2016). 10.1186/s13046-016-0433-9 PMC5037895 27670139

[62] Ogitani Y, Aida T, Hagihara K, Yamaguchi J, Ishii C, Harada N (2016). DS-8201a, a novel HER2-targeting ADC with a novel DNA topoisomerase I inhibitor, demonstrates a promising antitumor efficacy with differentiation from T-DM1. Clin. Cancer Res..

[63] Iwata H, Tamura K, Doi T, Tsurutani J, Modi S, Park H (2018). Trastuzumab deruxtecan (DS-8201a) in subjects with HER2-expressing solid tumors: Long-term results of a large phase 1 study with multiple expansion cohorts. J. Clin. Oncol..

[64] Doi T, Shitara K, Naito Y, Shimomura A, Fujiwara Y, Yonemori K (2017). Safety, pharmacokinetics, and antitumour activity of trastuzumab deruxtecan (DS-8201), a HER2-targeting antibody–drug conjugate, in patients with advanced breast and gastric or gastro-oesophageal tumours: a phase 1 dose-escalation study. Lancet Oncol..

[65] Tamura K, Tsurutani J, Takahashi S, Iwata H, Krop IE, Redfern C (2019). Trastuzumab deruxtecan (DS-8201a) in patients with advanced HER2-positive breast cancer previously treated with trastuzumab emtansine: a dose-expansion, phase 1 study. Lancet Oncol..

[66] Shitara K, Iwata H, Takahashi S, Tamura K, Park H, Modi S (2019). Trastuzumab deruxtecan (DS-8201a) in patients with advanced HER2-positive gastric cancer: a dose-expansion, phase 1 study. Lancet Oncol..

[67] Elgersma RC, Coumans RGE, Huijbregts T, WMPB Menge, JAF Joosten, Spijker HJ (2015). Design, synthesis, and evaluation of linker-duocarmycin payloads: Toward selection of HER2-targeting antibody-drug conjugate SYD985. Mol. Pharm..

[68] van der Lee MMC, Groothuis PG, Ubink R, van der Vleuten MAJ, van Achterberg TA, Loosveld EM (2015). The preclinical profile of the duocarmycin-based HER2-targeting ADC SYD985 predicts for clinical benefit in low HER2-expressing breast cancers. Mol. Cancer Ther..

[69] Menderes G, Bonazzoli E, Bellone S, Black J, Predolini F, Pettinella F (2017). SYD985, a novel duocarmycin-based HER2-targeting antibody-drug conjugate, shows antitumor activity in uterine and ovarian carcinosarcoma with HER2/Neu expression. Clin. Cancer Res..

[70] Banerji U, van Herpen CML, Saura C, Thistlethwaite F, Lord S, Moreno V (2019). Trastuzumab duocarmazine in locally advanced and metastatic solid tumours and HER2-expressing breast cancer: a phase 1 dose-escalation and dose-expansion study. Lancet Oncol..

[71] Spring L, Goel S, Sutherland S, Supko JG, Juric D, Isakoff SJ (2019). Trastuzumab emtansine (T-DM1) and ribociclib, an oral inhibitor of cyclin dependent kinase 4 and 6 (CDK 4/6), for patients with metastatic HER2-positive breast cancer. J. Clin. Oncol..

[72] Brandão M, Pondé NF, Poggio F, Kotecki N, Salis M, Lambertini M (2018). Combination therapies for the treatment of HER2-positive breast cancer: current and future prospects. Expert Rev. Anticancer Ther..

[73] Jain S, Shah AN, Santa-Maria CA, Siziopikou K, Rademaker A, Helenowski I (2018). Phase I study of alpelisib (BYL-719) and trastuzumab emtansine (T-DM1) in HER2-positive metastatic breast cancer (MBC) after trastuzumab and taxane therapy. Breast Cancer Res. Treat..

[74] Gottesman, M. M., Pastan, I. H. The role of multidrug resistance efflux pumps in cancer: revisiting a JNCI publication exploring expression of the MDR1 (P-glycoprotein) gene. *J. Natl Cancer Inst.* **107**, pp. djv222 (2015). 10.1093/jnci/djv222 PMC4836801 26286731

